# Supplementary material for: Effects of Sesamin, the Major Furofuran Lignan of Sesame Oil, on the Amplitude and Gating of Voltage-Gated Na+ and K+ Currents
Source: Molecules. 2020 Jul 4;25(13):3062. doi: 10.3390/molecules25133062 (PMC7411736; doi:10.3390/molecules25133062)
Supplement: Supplementary file 1 [file molecules-25-03062-s001.pdf]

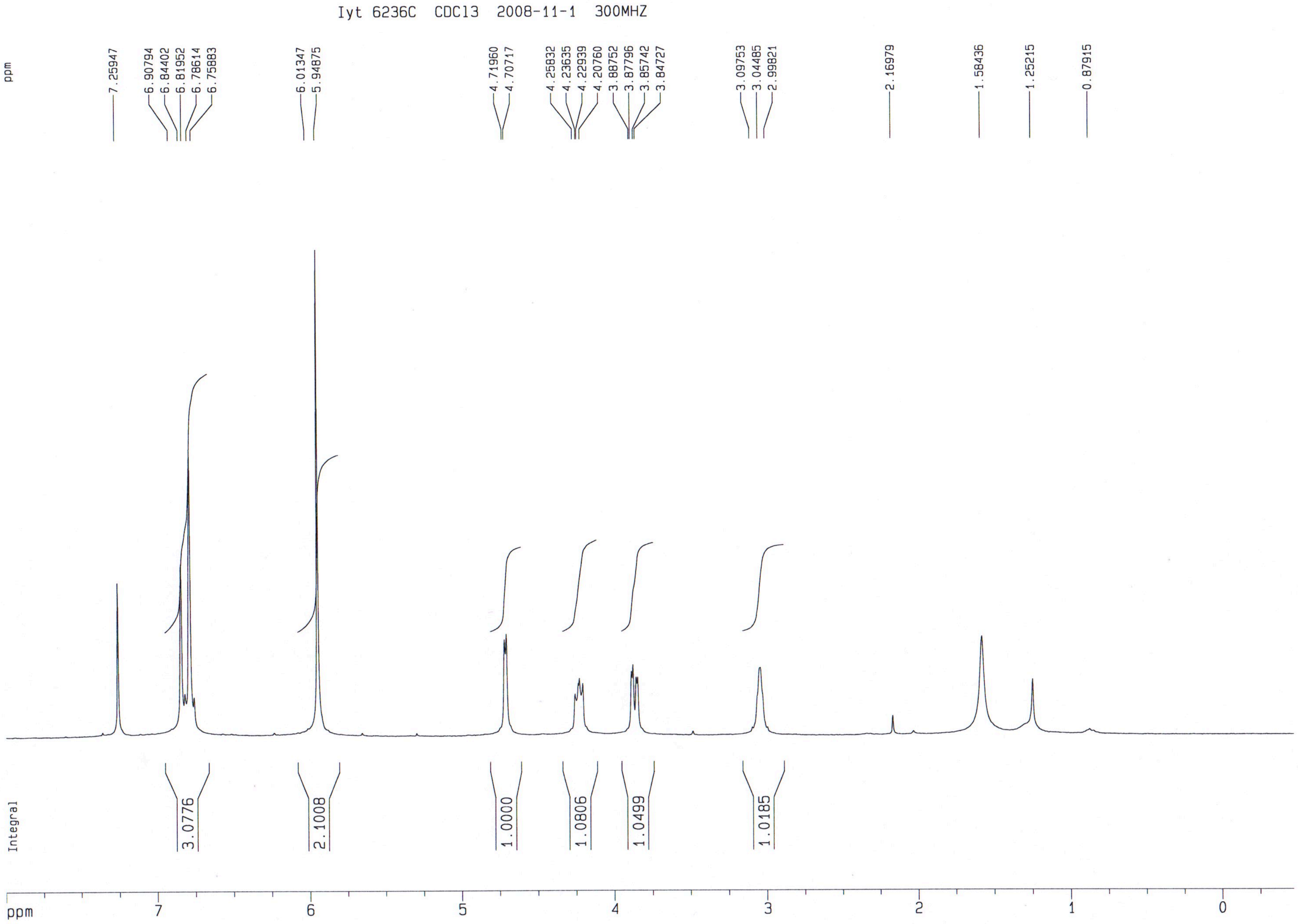
Iyt 6236C CDCl3 2008-11-1 300MHZ
ppm
7.25947
6.90794
6.84402
6.81952
6.78614
6.75883
6.01347
5.94875
4.71960
4.70717
4.25832
4.23635
4.22939
4.20760
3.88752
3.87796
3.85742
3.84727
3.09753
3.04485
2.99821
2.16979
1.58436
1.25215
0.87915
Integral
3.0776
2.1008
1.0000
1.0806
1.0499
1.0185
7
6
5
4
3
2
1
0
ppm

Sesamin

Agilent HC-C8, 5 μm , 4.6*250 mm , 0.5 mL/min

MeOH : H_2O = 65 : 35

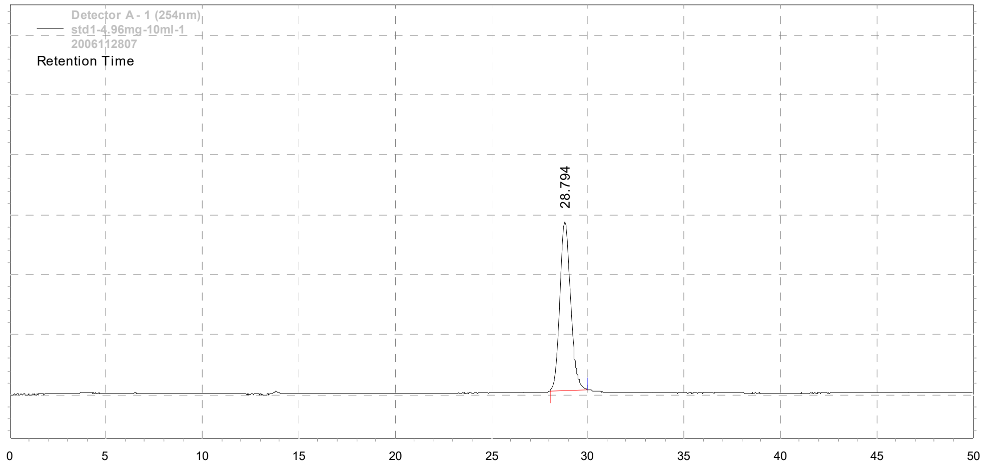

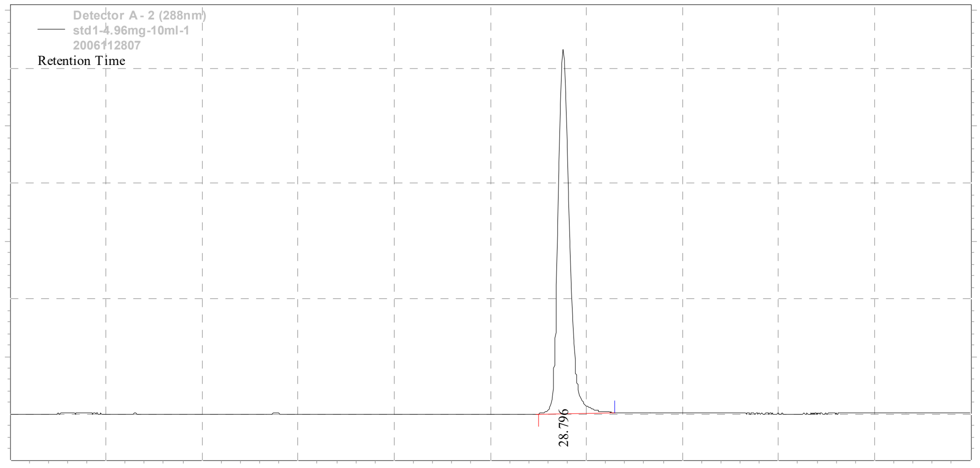

NO.0015,100916,1136, ,ROTATION=
+0.07,ISS=+0.21,ISS(TC)=+0.21,S.R.=+70.0
0,CONC=0.11,PURITY=0.42,POL=0.10,TEMP=27
.0,L=100,WAVELENGTH=589,ATAGO AP-300,S/N
= ,USER ID= ,USER NAME
= ,

NO.0016,100916,1137, ,ROTATION=
+0.13,ISS=+0.37,ISS(TC)=+0.37,S.R.=+130.
00,CONC=0.20,PURITY=0.76,POL=0.18,TEMP=2
7.0,L=100,WAVELENGTH=589,ATAGO AP-300,S/
N= ,USER ID= ,USER NAM
E= ,

NO.0017,100916,1138, ,ROTATION=
+0.07,ISS=+0.21,ISS(TC)=+0.21,S.R.=+70.0
0,CONC=0.11,PURITY=0.42,POL=0.10,TEMP=26
.9,L=100,WAVELENGTH=589,ATAGO AP-300,S/N
= ,USER ID= ,USER NAME
= ,

NO.0018,100916,1139, ,ROTATION=
+0.07,ISS=+0.21,ISS(TC)=+0.21,S.R.=+70.0
0,CONC=0.11,PURITY=0.42,POL=0.10,TEMP=26
.9,L=100,WAVELENGTH=589,ATAGO AP-300,S/N
= ,USER ID= ,USER NAME
= ,

NO.0019,100916,1140, ,ROTATION=
+0.08,ISS=+0.23,ISS(TC)=+0.23,S.R.=+80.0
0,CONC=0.12,PURITY=0.46,POL=0.11,TEMP=26
.8,L=100,WAVELENGTH=589,ATAGO AP-300,S/N
= ,USER ID= ,USER NAME
= ,

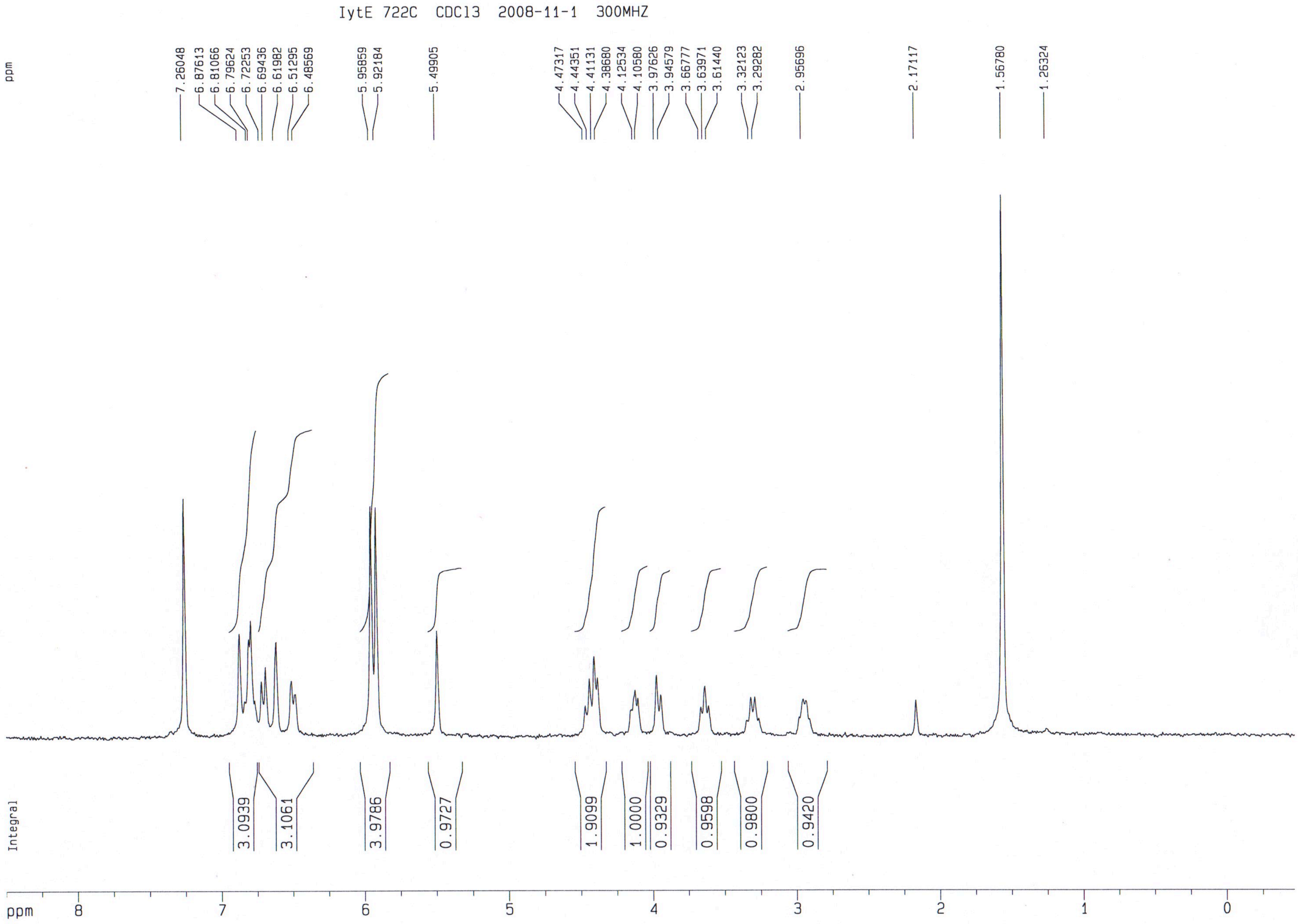
IytE 722C CDCl3 2008-11-1 300MHZ
ppm
7.26048
6.87613
6.81066
6.79624
6.72253
6.69436
6.61982
6.51295
6.48569
5.95859
5.92184
5.49905
4.47317
4.44351
4.41131
4.38680
4.12534
4.10580
3.97626
3.94579
3.66777
3.63971
3.61440
3.32123
3.29282
2.95696
2.17117
1.56780
1.26324
Integral
3.0939
3.1061
3.9786
0.9727
1.9099
1.0000
0.9329
0.9598
0.9800
0.9420
ppm 8 7 6 5 4 3 2 1 0

Sesamolin

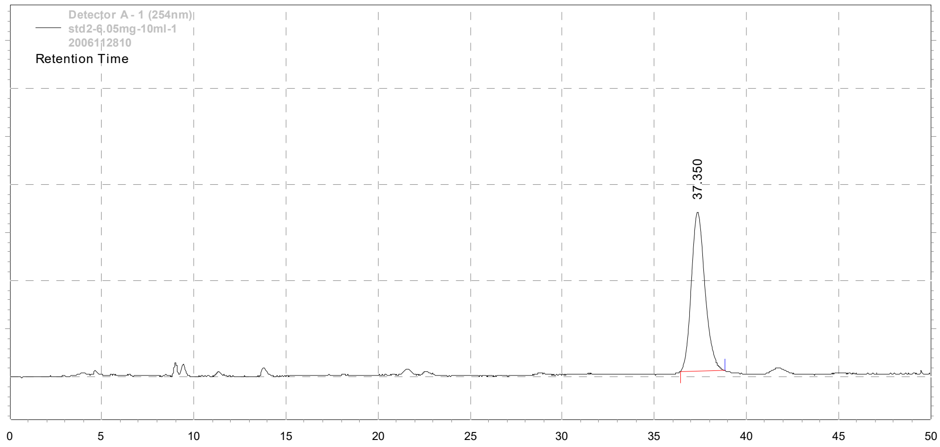
Detector A - 1 (254nm)
std2-6.05mg-10ml-1
2006112810
Retention Time
37.350
0
5
10
15
20
25
30
35
40
45
50

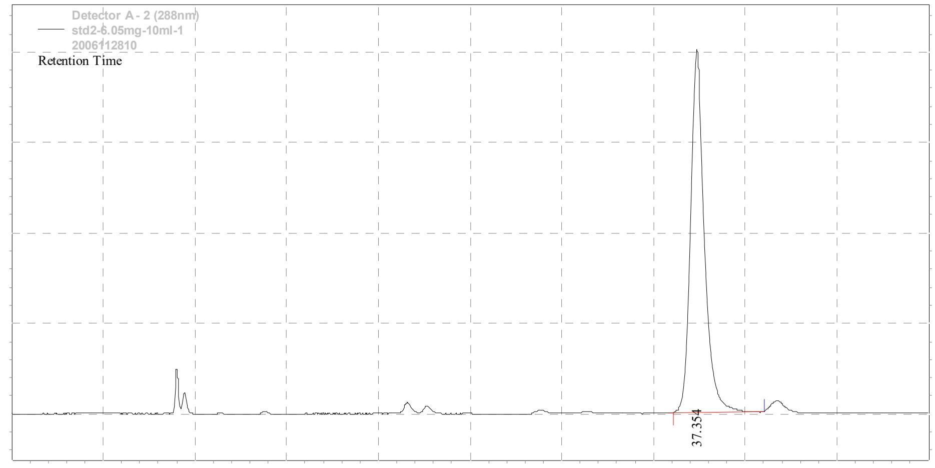
Detector A - 2 (288nm)
std2-6.05mg-10ml-1
2006112810
Retention Time
37.354

NO.0006,100916,1118,   ,ROTATION=
+0.18,ISS=+0.52,ISS(TC)=+0.52,S.R.=+180.
00,CONC=0.27,PURITY=1.05,POL=0.25,TEMP=2
6.8,L=100,WAVELENGTH=589,ATAGO AP-300,S/
N=   ,USER ID=   ,USER NAM
E=   ,

NO.0007,100916,1119,   ,ROTATION=
+0.20,ISS=+0.57,ISS(TC)=+0.57,S.R.=+200.
00,CONC=0.30,PURITY=1.14,POL=0.27,TEMP=2
7.0,L=100,WAVELENGTH=589,ATAGO AP-300,S/
N=   ,USER ID=   ,USER NAM
E=   ,

NO.0008,100916,1120,   ,ROTATION=
+0.19,ISS=+0.56,ISS(TC)=+0.56,S.R.=+190.
00,CONC=0.29,PURITY=1.14,POL=0.27,TEMP=2
7.1,L=100,WAVELENGTH=589,ATAGO AP-300,S/
N=   ,USER ID=   ,USER NAM
E=   ,

NO.0009,100916,1121,   ,ROTATION=
+0.21,ISS=+0.60,ISS(TC)=+0.60,S.R.=+210.
00,CONC=0.32,PURITY=1.18,POL=0.28,TEMP=2
7.3,L=100,WAVELENGTH=589,ATAGO AP-300,S/
N=   ,USER ID=   ,USER NAM
E=   ,

NO.0010,100916,1122,   ,ROTATION=
+0.22,ISS=+0.64,ISS(TC)=+0.64,S.R.=+220.
00,CONC=0.33,PURITY=1.27,POL=0.30,TEMP=2
7.5,L=100,WAVELENGTH=589,ATAGO AP-300,S/
N=   ,USER ID=   ,USER NAM
E=   ,
